# Gallstone Ileus With Atypical Gastrointestinal Bleeding: A Case Report and Literature Review

**DOI:** 10.7759/cureus.109974

**Published:** 2026-05-31

**Authors:** Armando Viramontes, Mario Alberto Munoz Flores, Carlos Javier Mares Jimenez, Eduardo Enrique Soto Ruiz, Jazmin Baca-Moreno

**Affiliations:** 1 General Surgery, Clinic Hospital Institute of Security and Social Services for State Workers, Ensenada, MEX; 2 Radiology, Mexicali General Hospital, Mexicali, MEX; 3 General Surgery, Red Cross Tijuana, Tijuana, MEX; 4 General Surgery, Mexican Social Security Institute (IMSS) Hospital General Regional No 1 Tijuana, Tijuana, MEX

**Keywords:** elderly, enterolithotomy, gallstone ileus, intestinal obstruction, rigler’s triad

## Abstract

Gallstone ileus is a rare complication of cholelithiasis. Its diagnosis is challenging due to a nonspecific presentation and high morbidity and mortality related to age and comorbidities. An 87-year-old female with a history of hypertension presented with obstructive abdominal pain, hyporexia, and "coffee-ground" emesis. Upon admission, she showed hemodynamic instability [blood pressure (BP) 83/51 mmHg] and acute renal failure [Creatinine (Cr) 1.34 mg/dL]. Computed tomography revealed Rigler's triad, consisting of small bowel obstruction, neumobilia, and an ectopic gallstone. Exploratory laparotomy identified a stone impacted 140 cm from the Treitz ligament. A "milking" maneuver and simple enterolithotomy were performed, extracting a 2.5 cm gallstone. The patient had a favorable recovery and was discharged on the sixth postoperative day. Gallstone ileus should be suspected in geriatric patients with obstructive symptoms, even when presenting with atypical gastrointestinal bleeding. In extremely frail or hemodynamically unstable patients, simple enterolithotomy remains the gold standard to reduce surgical time and intraoperative complications.

## Introduction

Gallstone ileus is a mechanical obstruction of the digestive tract caused by the migration of a biliary stone through a cholecystoenteric fistula, most commonly cholecystoduodenal [1]. Although it is an infrequent complication of cholelithiasis, representing only 0.3% to 0.5% of cases, its incidence increases significantly in the geriatric population, accounting for up to 25% of small bowel obstructions in patients over 65 years of age [2-3]. Managing this condition remains a surgical challenge as it predominantly affects elderly patients with multiple comorbidities, leading to reported mortality rates between 7% and 30% [3].

The clinical presentation is often insidious, characterized by the "tumbling obstruction" sign, where the stone impacts and disimpacts intermittently before definitive occlusion occurs [4]. However, atypical presentations may occur; erosion of the gastrointestinal mucosa during fistula formation or stone passage can cause upper gastrointestinal bleeding, manifested as hematemesis or melena, potentially diverting the initial diagnostic focus toward peptic or neoplastic pathologies [4-5].

Preoperative diagnosis has been optimized by computed tomography (CT), which allows the identification of Rigler's triad: pneumobilia, small bowel obstruction, and an ectopic gallstone [6]. The sensitivity of this study exceeds 90%, making it the current gold standard [6-8]. Regarding treatment, the debate between simple enterolithotomy and one-stage surgery (which includes cholecystectomy and fistula closure) persists [9]. Nevertheless, in scenarios of hemodynamic instability or extreme frailty, simple enterolithotomy is positioned as the safest option to reduce perioperative morbidity and mortality [9].

We present the case of an 87-year-old female with gallstone ileus and an atypical clinical presentation involving gastrointestinal bleeding, emphasizing the importance of clinical suspicion and simplified surgical management in the octogenarian patient.

## Case presentation

Patient information and history

The patient was an 87-year-old female with a recently diagnosed history of systemic arterial hypertension, treated with angiotensin-converting enzyme (ACE) inhibitors. Her surgical background included a total abdominal hysterectomy performed 40 years ago for uterine myomatosis. She denied chronic use of non-steroidal anti-inflammatory drugs (NSAIDs) or previous similar episodes.

Clinical presentation and initial evaluation

Her illness began two weeks before admission with colicky abdominal pain (10/10 on the visual analog scale), located in the left quadrants and radiating to the right flank. Five days before hospitalization, she developed coffee-ground emesis, hyporexia, and melena. She also reported a one-month change in bowel habits (Bristol three every 48 hours) and unquantified weight loss. Upon arrival at the emergency department, the patient presented with hemodynamic compromise and signs of cellular hypoperfusion: blood pressure (BP) 83/51 mmHg, heart rate 111 bpm, respiratory rate 18 rpm, temperature 35.5 °C, and 96% oxygen saturation on room air (Table 1). Physical examination showed a flat abdomen with hyperactive peristalsis and pain on deep palpation in the left iliac fossa and flank, without signs of peritoneal irritation.

**Table 1:** Initial Vital Signs mmHg: millimeters of mercury; bpm: beats per minute; rpm: respirations per minute; °C: degrees Celsius; %: percentage.

Parameter		Patient Value		Reference Range	
Blood pressure		83/51 mmHg		120/80 mmHg	
						Heart rate		111 bpm		60–100 bpm	
												Respiratory rate		18 rpm		12–20 rpm	
Body temperature		35.5 °C		36.5–37.2 °C													
						Oxygen saturation		96%		95–100% (on room air)							

Diagnostic assessment

Laboratory results indicated a hemoglobin level of 10.7 g/dL (moderate anemia), normal leukocyte counts, and an elevated erythrocyte sedimentation rate (ESR) of 85 mm/hr. The biochemical profile showed a creatinine (Cr) of 1.34 mg/dL (suggesting prerenal acute kidney injury) and direct hyperbilirubinemia (1.97 mg/dL). Electrolytes and arterial blood gas were within normal ranges (Table 2).

**Table 2:** Initial Laboratory Results g/dL: grams per deciliter; /µL: per microliter; mm/hr: millimeters per hour; mg/dL: milligrams per deciliter; mEq/L: milliequivalents per liter.

Parameter		Patient Value		Reference Range	
Hemoglobin		10.7 g/dL		12.0–15.5 g/dL	
						Leukocyte count		9,520 /µL		4,500–11,000 /µL	
												Erythrocyte sedimentation rate		85 mm/hr		0–30 mm/hr	
Serum creatinine		1.34 mg/dL		0.5–1.1 mg/dL													
						Direct bilirubin		1.97 mg/dL		0.0–0.3 mg/dL							
												Sodium		137 mEq/L		135–145 mEq/L	
Potassium		4.5 mEq/L		3.5–5.1 mEq/L													
						Chloride		103 mEq/L		98–107 mEq/L							
												Calcium		9.0 mg/dL		8.5–10.2 mg/dL	

An abdominopelvic computed tomography (CT) with intravenous contrast was performed, which documented Rigler's triad: mechanical small bowel obstruction with dilated loops, pneumobilia (air in the biliary tract and gallbladder), and a 42 mm "target" image in the left flank with internal calcific densities, compatible with an ectopic gallstone (Figure 1).

**Figure 1:**
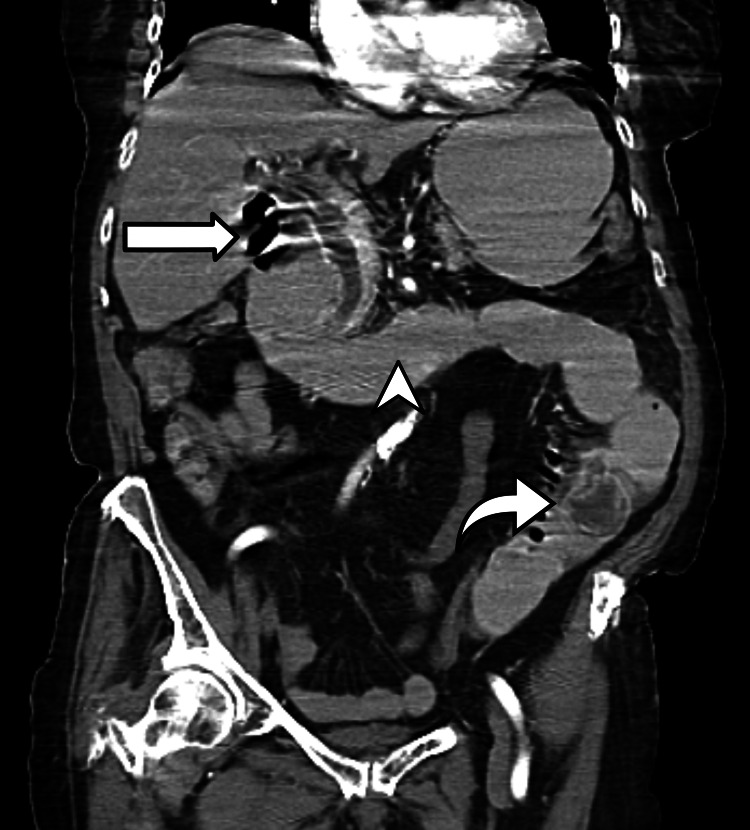
Abdominopelvic CT Scan With Intravenous Contrast Demonstrates Rigler's triad with findings of bowel loop dilation (arrowhead), pneumobilia (straight arrow), and ectopic gallstone (curved arrow).

Therapeutic intervention

Based on the diagnosis of gallstone ileus and hemodynamic instability, urgent surgical intervention was initiated. An exploratory laparotomy was performed through a 20 cm supra-infraumbilical midline incision. Cavity exploration revealed marked dilation of small bowel loops up to 140 cm from the ligament of Treitz, the site of impact for an intraluminal foreign body. A milking maneuver was performed to move the stone 20 cm proximally toward a segment with better trophic characteristics. A 2.5 cm longitudinal enterotomy was performed on the antimesenteric border, and a single gallstone approximately 2.5 cm in diameter was extracted. (Figure 2) The intestinal wall was closed in two planes: a continuous transmural layer with 3-0 Vicryl and an invaginating second layer (Lembert sutures) with 2-0 silk, ensuring hermeticity. Systematic review identified the gallbladder with firm adhesions to the Hartmann's pouch, consistent with a cholecystoenteric fistula; however, given the patient's hemodynamic status and advanced age, no dissection or treatment of the fistula was performed.

**Figure 2:**
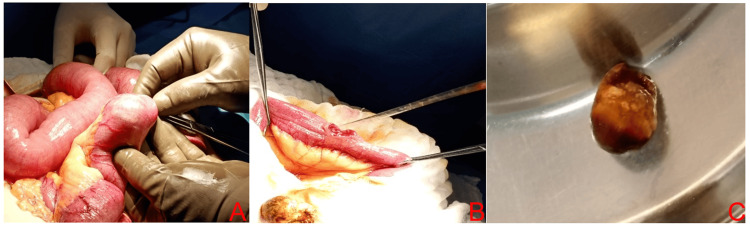
Intraoperative Imaging A. Demonstrates the milking maneuver to mobilize the stone into the healthy intestine. B. Demonstrates a longitudinal enterotomy. C. Gallstone of approximately 2.5 cm.

Follow-up and outcomes

The patient’s postoperative evolution was satisfactory. She began early ambulation and successfully progressed to an oral diet. She was discharged on the sixth postoperative day without any complications recorded during her hospital stay.

## Discussion

Epidemiology and clinical profile

Gallstone ileus is an uncommon complication of cholelithiasis, representing 0.3%-0.5% of cases; however, its clinical relevance lies in the fact that it accounts for up to 25% of mechanical obstructions in patients over 65 years of age [1-3]. In our 87-year-old patient, advanced age and a recent diagnosis of hypertension align with the high-risk demographic profile, where frailty conditions a reported mortality of up to 30% [6].

Atypical findings and diagnostic challenges

A distinctive finding in this case was coffee-ground emesis and melena. This presentation is uncommon and usually results from mucosal erosion during the formation of a cholecystogastric or duodenal fistula, or from trauma caused by the gallstone as it transits through the digestive tract [5,10-11]. This "clinical distractor" underscores the need to include gallstone ileus in the differential diagnosis of upper gastrointestinal bleeding in the elderly, especially when accompanied by signs of bowel obstruction.

Computed tomography (CT) allowed for the identification of Rigler's triad with high precision, which is the current diagnostic gold standard [4,7]. A fundamental technical aspect during surgery was the systematic review of the bowel down to the ileocecal valve. This maneuver is critical, as literature reports that up to 5%-16% of patients may have a synchronous second stone that is not initially detected, which would lead to persistent or recurrent obstruction if not extracted during the initial surgical procedure [7].

Surgical strategy: enterolithotomy versus one-stage surgery

The debate over whether to perform a simple enterolithotomy or a definitive procedure (enterolithotomy, cholecystectomy, and fistula closure) is central to surgical literature. While a laparoscopic approach is a valid option in stable patients and experienced centers [12], midline laparotomy remains preferable in the context of an emergency with hemodynamic compromise. In this patient, with a BP of 83/51 mmHg and acute renal failure (Cr 1.34 mg/dL), the priority was rapid resolution of the obstruction. Large-scale national studies demonstrate that simple enterolithotomy is associated with lower rates of major complications and lower mortality compared to one-stage surgery in patients with severe comorbidities or instability [9].

Strengths and limitations

The strengths of this report include the rapid identification of Rigler's triad via CT, which allowed for timely intervention and prevented progression to intestinal ischemia or perforation in an elderly patient. The decision to perform simplified surgery (enterolithotomy) aligned with current guidelines for managing hemodynamically unstable patients.

Regarding limitations, as this is a single case report, the results cannot be generalized. Additionally, long-term imaging follow-up was not performed to evaluate the spontaneous evolution of the persistent cholecystoenteric fistula, although literature suggests that many close spontaneously once biliary flow is normalized.

## Conclusions

Gallstone ileus should be maintained as a priority differential diagnosis in geriatric patients presenting with intestinal obstruction, even when atypical symptoms like upper gastrointestinal bleeding are present. Computed tomography stands as the definitive diagnostic tool, enabling the visualization of Rigler’s triad. In octogenarian patients with hemodynamic compromise, simple enterolithotomy combined with a "milking" maneuver remains the safest approach, prioritizing the resolution of the obstruction over biliary fistula repair to minimize perioperative morbidity and mortality.

The patient and her relatives expressed satisfaction with the rapid resolution of pain and the restoration of bowel function. They accepted the conservative management of the residual gallbladder pathology after being informed of the risks associated with more extensive surgical procedures.
